# Supplementary figures and images for: Use of pyriproxyfen in control of Aedes mosquitoes: A systematic review
Source: PLoS Negl Trop Dis. 2020 Jun 12;14(6):e0008205. doi: 10.1371/journal.pntd.0008205 (PMC7314096; doi:10.1371/journal.pntd.0008205)

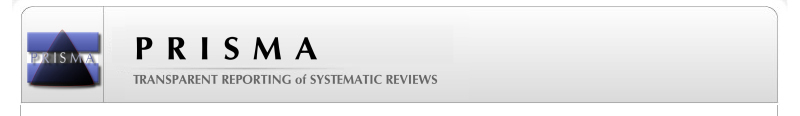
**PRISMA 2009 Flow Diagram**

Supplement: S1 Fig — (DOCX) [file pntd.0008205.s001.docx]
