# Supplementary material for: Use of pyriproxyfen in control of Aedes mosquitoes: A systematic review
Source: PLoS Negl Trop Dis. 2020 Jun 12;14(6):e0008205. doi: 10.1371/journal.pntd.0008205 (PMC7314096; doi:10.1371/journal.pntd.0008205)
Supplement: Attachment — Submitted filename: PNTD 11-18-19.docx [file pntd.0008205.s003.docx]

Use of pyriproxyfen in control of *Aedes* mosquitoes: a systematic review

Hustedt, J.

**GENERAL.**

Since a recent review of the effectiveness of pyriproxyfen (PPF), the current study have identified several other candidate studies to evaluate (17 studies in previous review of 2017 and 90 studies in current review). The review is comprehensive and I have provided some comments and suggestions for improvement.

**SPECIFIC.**

**Abstract.**

Line 15. *Aedes* is a large genus. Only a few species are vectors of human pathogens (throughout paper).

Line 17. Change “in capable” to “incapable.”

Line 18 “Emergence to adulthood” or “eclosion”

Lines 30-31. Does this suggest some type of cross-resistance?

Is it possible to make any recommendations for control strategies based on this studies findings?

**Author summary.**

Lines 44-46. This sentence suggests that the influence of PPF was also measured on adults (e.g., traits, behaviors). There is evidence for substantial effects of PPF on adults, but the abstract and cover letter do not suggest this information was assessed.

**Introduction.**

This section is very brief. Additional information would be useful. For example, Zika, Chikungunya, and yellow fever (Brazil) viruses have emerged in recent years. These viruses are transmitted primarily by *A. aegypti* and *A. albpictus,* as suggested in the author summary section.

**Methods.**

Table 1. All the outcome measures seem appropriate and comprehensive of information about vector abundance.

Database search. All this information seems appropriate.

Table 2. I suggest including the search term “autodissemination of pyriproxyfen”

Lines, 120-121. A meta-analysis would be appropriate to quantify the effects of PPF on mosquito suppression.

**Results.**

Lines 130-131. Please be more specific (e.g., No data on the effect PPF on mosquito suppression was report in these studies).

Lines 138-151. This is useful information regarding the specifics of the use of PPF.

Lines 155-162. Could you explain here (or Discussion) the discrepancies and effectiveness of PPF for suppression of mosquitoes observed between studies.

Line 179. Please be more specific “… in a reduction of mosquito population by juvenile mortality and subsequent recruitment of adults”

Lines 176-182. I suggest separating (or specifying) this sections results into both lab and field studies. Additional details on these studies would be useful.

Figures 1-4 appear to be tables, not figures.

Line 185. “… tested the theory…” Please use proper grammatical terms (What theory? Is what you are referring to an actual “theory”) and be specific on what is being described.

Lines 232-235. Please be specific on the stage of mosquito that was being measured for suppression following PPF exposure. That is, although PPF primarily induces mortality during the pupal stage, there are certainly effects on the adult stage that limit propagation of the adult population (as mentioned in subsequent sentences of this section).

Lines 235-238. Good description of effects on adult traits.

Lines 245-247. Spatial aspects of the effectiveness are more relevant than number of houses treated.

Lines 265-266. Specify citations.

Lines 274-276. Can you provide a potential mechanism for this observation?

**Discussion.**

Lines 292-298. Also, the efficacy of PPF may be altered for populations of mosquitoes known to be resistant to organophosphates (temephos).

Lines 306. Incomplete sentence.
