# Supplementary material for: Use of pyriproxyfen in control of Aedes mosquitoes: A systematic review
Source: PLoS Negl Trop Dis. 2020 Jun 12;14(6):e0008205. doi: 10.1371/journal.pntd.0008205 (PMC7314096; doi:10.1371/journal.pntd.0008205)
Supplement: Attachment — Submitted filename: Response to Reviewers.docx [file pntd.0008205.s004.docx]

Use of pyriproxyfen in control of *Aedes* mosquitoes: a systematic review

Hustedt, J.

**GENERAL.**

Since a recent review of the effectiveness of pyriproxyfen (PPF), the current study have identified several other candidate studies to evaluate (17 studies in previous review of 2017 and 90 studies in current review). The review is comprehensive and I have provided some comments and suggestions for improvement.

Thanks for commenting the review is comprehensive.

**SPECIFIC.**

**Abstract.**

Line 15. *Aedes* is a large genus. Only a few species are vectors of human pathogens (throughout paper).

Thanks for pointing this out. To be more precise we have added “principally *Aedes aegypti”* to the abstract on line 15 as below. This was also put in line 59 and 84.

*“means of controlling Aedes mosquitos, principally Aedes aegypti, as the main vector of dengue*

Line 17. Change “in capable” to “incapable.”

This has been changed

Line 18 “Emergence to adulthood” or “eclosion”

This has been changed

Lines 30-31. Does this suggest some type of cross-resistance?

Is it possible to make any recommendations for control strategies based on this studies findings?

Thanks for this question. It does suggest that there may be some cross-resistance. A further discussion of this is held in the results section starting on line 256 and the discussion section starting on line 321. The recommendation on line 356 was made was below:

*“Therefore, in areas where there is already increased resistance to PPF, control programs should consider combining insecticides that work in synergy. Regular entomological surveillance to monitor the susceptibility status of Aedes mosquitoes can help provide evidence and prevent development of resistance [83].”*

**Author summary.**

Lines 44-46. This sentence suggests that the influence of PPF was also measured on adults (e.g., traits, behaviors). There is evidence for substantial effects of PPF on adults, but the abstract and cover letter do not suggest this information was assessed.

Thanks for noticing this. In order to make this clearer and more consistent we have added the wording from the abstract. It now reads as below on line 45:

*“inhibition of emergence to adulthood, larval mortality, and resistance ratios”*

**Introduction.**

This section is very brief. Additional information would be useful. For example, Zika, Chikungunya, and yellow fever (Brazil) viruses have emerged in recent years. These viruses are transmitted primarily by *A. aegypti* and *A. albpictus,* as suggested in the author summary section.

Thanks for your suggestions. We have increased the introduction section and added a line about the additional viruses transmitted by *Aedes* mosquitoes on line 83 as follows:

*“In addition to reducing dengue these vector control methods can also reduce other diseases transmitted by Aedes mosquitoes such as chikungunya, Zika, and yellow fever.”*

**Methods.**

Table 1. All the outcome measures seem appropriate and comprehensive of information about vector abundance.

Database search. All this information seems appropriate.

Table 2. I suggest including the search term “autodissemination of pyriproxyfen”

Thanks for this suggestion. We have added this search term but included it as (autodissemination of pyriproxyfen) AND *Aedes*. Please see the following table with the results (and the flow chart in the MS):

Table 1: Papers found with additional search term “autodissemination of pyriproxyfen”

| Database | # Identified | duplicates | eliminated by title | eliminated by abstract | eliminated by full text | included |
| --- | --- | --- | --- | --- | --- | --- |
| Embase | 0 | 0 | 0 | 0 | 0 | 0 |
| web of science | 18 | 10 | 6 | 2 | 0 | 0 |
| LILACS | 19 | 18 | 0 | 0 | 1 | 0 |
| Global health | 1 | 1 | 0 | 0 | 0 | 0 |
| Cochrane Database of Systematic Reviews | 0 | 0 | 0 | 0 | 0 | 0 |
| Pubmed | 19 | 19 | 0 | 0 | 0 | 0 |
| Total | 57 | 48 | 6 | 2 | 1 | 0 |

Two were eliminated on the basis of the abstract because there was no auto-dissemination data included or they were meeting notes. One was excluded after reading the full text because there was no original data. These additional results have been added into the flow chart and in the search results section on line 186.

Lines, 120-121. A meta-analysis would be appropriate to quantify the effects of PPF on mosquito suppression.

Thanks for this suggestion. We also would have liked to include a meta-analysis. However, the heterogeneity of the studies would make the interpretation very difficult. For example, the majority of studies were related to PPF granules, and, within these, the most frequent product used was Sumilarv 0.5G (16 studies). However, within these studies, the dosage ranged quite a bit from 0.002 to 100 PPM. There was also only one semi-field study and three field studies. None of the field studies used all the same concentrations (one study did not even report the concentration). Turning to the lab studies, they also had a very wide range of concentrations under varying conditions. So again, we concluded that a meta-analysis would be misleading. In the discussion section on line 372 we have explained these reasons for not doing meta-analyses and recognized this as a gap in current knowledge.

**Results.**

Lines 130-131. Please be more specific (e.g., No data on the effect PPF on mosquito suppression was report in these studies).

We have tried to be more specific here on line 150:

“The most common reasons for exclusion among the full text articles were that papers on PFF reported non-original data, e.g. reviews or perspective pieces (n=10), or PPF was only tangentially mentioned (again with no relevant original data, n=8).”

Lines 138-151. This is useful information regarding the specifics of the use of PPF.

Thanks for thinking this was useful information.

Lines 155-162. Could you explain here (or Discussion) the discrepancies and effectiveness of PPF for suppression of mosquitoes observed between studies.

Thanks for this question. In fact, our interpretation is that the picture is generally consistent in that higher concentrations of PPF granules were generally effective. I have added the following in the main text on line 176:

“Despite some discrepancies, most studies showed Inhibition of Emergence (IE) near 100% for 90 days at higher concentrations (1-10 parts-per-million (ppm)), and a steady reduction with time post-treatment or with decreasing concentration of active ingredient (Fig 2).”

Line 179. Please be more specific “… in a reduction of mosquito population by juvenile mortality and subsequent recruitment of adults”

Thanks for this suggestion. We have attempted to be more specific as you suggested.

Lines 176-182. I suggest separating (or specifying) this sections results into both lab and field studies. Additional details on these studies would be useful.

Thanks for this suggestion, which we have followed.

Figures 1-4 appear to be tables, not figures.

Thank you for noticing this. It was difficult for me to insert the tables directly into the text, and therefore was trying to follow the author guidelines in the tables section: <https://journals.plos.org/plosone/s/tables>.

**“When should I make my table a figure?**
If the table has a very complex structure or contains graphics, the safest solution is to make it into a figure. Export the table as a TIFF, and cite and re-label it as a figure.”

Line 185. “… tested the theory…” Please use proper grammatical terms (What theory? Is what you are referring to an actual “theory”) and be specific on what is being described.

Thanks for picking this up. This has now been reworded (line 254).

Lines 232-235. Please be specific on the stage of mosquito that was being measured for suppression following PPF exposure. That is, although PPF primarily induces mortality during the pupal stage, there are certainly effects on the adult stage that limit propagation of the adult population (as mentioned in subsequent sentences of this section).

Thanks for this suggestion. I have tried to do as you suggested on line 312:

“Beyond simply having an effect on larval mortality, the sublethal dose of PPF was found to have effects on the fertility and fecundity of adult females who were exposed at the adult stage.”

Lines 235-238. Good description of effects on adult traits.

Lines 245-247. Spatial aspects of the effectiveness are more relevant than number of houses treated.

Thanks for pointing this out. I looked in the paper and they didn’t say exactly how big or spread out the area was. They did say the houses were the same size and evenly spaced in the area. Therefore, I added this on line 322 as follows:

(200 houses the same size and evenly spaced and distributed within each of three different treatment areas)

Lines 265-266. Specify citations.

Thanks. I have tried to do this on line 490

Lines 274-276. Can you provide a potential mechanism for this observation?

Thanks for suggesting adding this. I have tried to do so on line 456:

“Significant differences in detoxification enzyme activities in several resistant *Aedes albopictus* populations in the USA suggest the involvement of a metabolic based resistance mechanism [86].”

**Discussion.**

Lines 292-298. Also, the efficacy of PPF may be altered for populations of mosquitoes known to be resistant to organophosphates (temephos).

Thanks for this point. I have included it on line 477.

Lines 306. Incomplete sentence.

Thanks for catching that. I have tried to finish the sentence on line 488 as follows:

“and it is still low enough that additional tools may need to be used in combination with PPF to reduce *Aedes* populations to zero (e.g. granules or controlled release devices for key containers).“

Reviewer #1 comments:

Reviewer #1: For the methods section, please highlight better the inclusion criteria and add exclusion criteria.

Thanks for this suggestion. I have tried to do this on line 197 as follows:

“All studies reporting original data on the use of pyriproxyfen in control of *Aedes or Stegomyia* as a single agent or combined with other control measures were eligible for inclusion. Any study not meeting the inclusion criteria above was excluded.”

Please add how the analysis of the massive amount of data from 91 papers was conducted.

This was done on line 223 as follows:

“The data extraction sheet was used to enter relevant information on each of the included studies, and the resulting tables were used to analyse the effect of PPF. Additional relevant information from each of the studies was entered in a comment section of the extraction sheet for further analysis when writing the manuscript.”

Was a quality assessment of the studies done? This needs to be added.

Thanks for this suggestion. Strengths and weaknesses for each of the studies in the original extraction was done and added to the tables. However, this resulted in extremely long tables and the authors felt it would be hard for readers to absorb that much data. To keep the tables readable and interpretable this information was not included in the tables.

Reviewer #1: For the descriptive part of the results section, please add numbers for the exclusion of papers.

Thanks for this suggestion. The numbers for exclusion of papers have been edited starting on line 230 as follows:

“Initially 1,409 records were identified through database searches and 17 additional records were identified through other sources. After screening of title and abstracts, the remaining 109 papers were assessed and reviewed in full, after which 18 articles were excluded.”

An analysis by authors/groups of authors would be useful, please add.

Thanks for this suggestion. As suggested above and in line with this comment we have made an effort to include or group where possible authors by study type (e.g. field, semi-field, or lab studies). In addition, in places where one author wrote multiple manuscripts, we have made an effort to group them. However, in the case of auto-dissemination and ULV treatments most of the authors who wrote multiple papers did so publishing the lab studies first and then confirmatory field studies second. Therefore, we had to choose if we would follow the above suggestions of the reviewer to group by study type or by author. In those cases we chose to group by study type since we felt it helped the flow of the manuscript.

Reviewer #1: Figures in the tables are corresponding to the references for figures in the text? E.g. figure 3 is referenced in the text in line 182, and figure 2 follows in line 183?

I believe this is answered by the response to the reviewer above. Please see the answer to that question above.

Figure 1,3,4: please add a column about the study design, with the key characteristics. As done in figure 2.

Thanks for this suggestion. If I’m not mistaken all the figures had a column about study design titled “Type of Study”.

PRISMA flowchart: please add some boxes to help reading the flowchart

Thanks for this suggestion. I have tried to make sure the boxes can be seen in this version.
